# Role of the prostaglandin E_2_ receptor agonists in TGF-β1-induced mesangial cell damage

**DOI:** 10.1042/BSR20160038

**Published:** 2016-09-29

**Authors:** Pei-pei Xi, Yu-yin Xu, Xiao-lan Chen, Ya-ping Fan, Jian-hua Wu

**Affiliations:** * Department of Nephrology, Affiliated Hospital of Nantong University, Nantong, 226001, Jiangsu, China; † Department of Emergency, Affiliated Hospital of Nantong University, Nantong, 226001, Jiangsu, China

**Keywords:** agonist, MAPKs signalling pathway, mesangial cells, prostaglandin E_2_ receptors, TGF-β1

## Abstract

PGE_2_ exerts its biological effect through binding to various EP receptors that result inactivation of various signal transduction pathways. It also plays an important role in mice glomerular mesangial cells (MCs) damage induced by transforming growth factor-β1 (TGF-β1); however, the molecular mechanisms remain unknown. In the present study, we tested the efficacy of four selective agonists of PGE_2_ receptor, EP_1_A (17-phenyl trinor prostaglandin E_2_ ethyl amid), EP_2_A (butaprost), EP_3_A (sulprostone) and EP_4_A (cay10580), on mice MCs. Compared with the cAMP produced by TGF-β1, additional pretreatment of EP_3_A decreased the cAMP level. MCs treated with EP_1_A and EP_3_A augmented PGE_2_, cyclooxygenase-2 (COX-2), membrane-bound PGE synthase 1 (mPGES1), laminin (LN), connective tissue growth factor (CTGF) and cyclin D1 expression stimulated by TGFβ1. EP_1_A and EP_3_A increased the number of cells in S+G2/M phase and reduced cells in G0/G1 phase. EP_1_ and EP_3_ agonists also strengthened TGFβ1-induced mitogen-activated protein kinase (p38MAPK) and extracellular-signal-regulated kinase 1/2 (ERK1/2) phosphorylation. Whereas MCs treated with EP_2_A and EP_4_A weakened PGE_2_, COX-2, mPGES1, LN, CTGF and cyclin D1 expression stimulated by TGFβ1. EP_2_A and EP_4_A decreased the number of cells in S+G2/M phase and increased cells in G0/G1 phase. EP_2_ and EP_4_ agonists weakened TGFβ1-induced p38MAPK and ERK1/2 phosphorylation. These findings suggest that PGE_2_ has an important role in the progression of kidney disease via the EP_1_/EP_3_ receptor, whereas EP_2_ and EP_4_ receptors are equally important in preserving the progression of chronic kidney failure. Thus, agonists of EP_2_ and EP_4_ receptors may provide a basis for treating kidney damage induced by TGF-β1.

## INTRODUCTION

Renal fibrosis, characterized by glomerulosclerosis and tubulointerstitial fibrosis, is the final common manifestation of a wide variety of chronic kidney diseases (CKD). CKD can be caused by a variety of factors, including diabetes, hypertension, infections, atherosclerosis, renal artery and ureteral obstruction and genetic alterations [1]. TGF-β has been recognized as an important mediator in the genesis of CKD, which is characterized by the accumulation of extracellular matrix (ECM) components in the glomeruli (glomerular fibrosis, glomerulosclerosis) and the tubular interstitium (tubulointerstitial fibrosis). Extensive studies have demonstrated that TGF-β induces mesangial expansion caused by mesangial cells (MCs) hypertrophy, proliferation (and eventually apoptosis) and ECM synthesis [2]. Accumulating evidences have suggested that eicosanoids derived from cyclooxygenase-2 (COX-2) and PGE_2_ participate in a number of pathological processes in immune-mediated renal diseases [3]. PGE_2_ has been shown to have multiple biological effects in the kidney including the regulation of vascular smooth muscle tonus, glomerular filtration, renin release and tubular salt and water transport. Similarly, in disease states such as diabetic nephropathy, PGE_2_ synthesis is elevated [4]. However, the biologic consequences of increased glomerular PGE_2_ synthesis under pathological environment remain poorly understood and worth our further study. Kreisberg found that MCs from diabetic rats and normal MCs cultured under high-glucose conditions produced significantly greater amounts of PGE_2_ than control MCs. Increased PGE_2_ production occurred concomitant with an increase in MCs proliferation [5]. Rodríguez-Barbero et al. [3] also demonstrated that TGF-β1 induced production of PGE_2_ through the activation of MAPK signalling pathway, which was followed by increased proliferation of MCs.

The pharmacological activity of PGE_2_ is carried out via four different cell surface receptor subtypes: EP_1_, EP_2_, EP_3_ and EP_4_ [6–9]. All the four EP receptors have been localized in the kidney. EP_2_ and EP_4_ receptors couple to Gs, and activation of these receptors results in stimulation of adenylate cyclase, increases intracellular cAMP and activation of protein kinase A (PKA) [10–12]. However, EP_2_ and EP_4_ receptor will play a different role in some physiological or pathophysiological process. Stimulation of the EP_1_ receptor results in activation of phosphatidylinositol (PI) hydrolysis and elevation of intracellular Ca^2+^ concentration [4,5]. The major signalling pathway described for the EP_3_ receptor is mediated by Gi and leads to a reduction in intracellular cAMP levels. But further studies have shown that certain splice variants of EP_3_ could activate cAMP production and promote the effect of IP_3_ [6,13]. In order to explore the different roles of the four receptors, we first identified the expression of EP_1_–EP_4_ by RT-PCR, and then used agonists of EP_1_, EP_2_, EP_3_ and EP_4_ to assess their effects on TGF-β1-induced cell damage and whether the regulation involves cAMP and MAPKs pathways.

## MATERIALS AND METHODS

### Materials

Murine MCs were kindly provided by donors Fudan University; cAMP and PGE_2_ ELISA kits were obtained from R&D; TGF-β1 was obtained from Peprotech; 17-phenyl PGE_2_ (EP_1_A, EP_1_ agonist, prostanoid receptor agonist selectivity: EP_1_>EP_3_), butaprost (EP_2_A, EP_2_ agonist), sulprostone (EP_3_A, prostanoid receptor agonist selectivity: EP_3_>EP_1_) and cay10580 (EP_4_A, EP_4_ agonist) were obtained from Cayman; Rabbit anti-phospho-p38 MAPK (Thr-180/Tyr-182), phospho-ERK1/2 (Thr-202/Tyr-204), phospho-JNK1/2 (T183/Y185), total JNK, total ERK1/2, total p38, p27kip1, cyclin D1, COX-2 were obtained from Cell Signaling Technology. Mouse anti-CCN2/connective tissue growth factor (CTGF), membrane-bound PGE synthase 1 (mPGES1), laminin (LN) were obtained from Abcam; Dulbecco's modified Eagle's medium, phosphate-buffered saline, trypsin, non-essential amino acids and antibiotics (penicillin/streptomycin) were obtained from Invitrogen; all solutions and instruments for RT-PCR were obtained from Applied Biosystems; 100-mm and 6-well cell culture plates were obtained from Fisher. The experimental protocol was approved by the Experimentation Ethics Committee of the Nantong University for the use of human or animal subjects.

### Cell culture

Mouse MCs were cultured in Dulbecco's modified Eagle's medium supplemented with 0.1 mM non-essential amino acids, 10% foetal bovine serum (FCS), 100 units/ml penicillin and 0.1 mg/ml streptomycin at 37°C in a humidified incubator containing 5% CO_2_. Cells were grown to 80% confluence and then rendered quiescent 24 h before experiment by the removal of serum and growth factor additives. Specified agonists were next added in serum-free bovine serum albumin medium for 30 min prior to the addition of TGF-β1 (10 ng/ml). Based on our pre-experimental results, the optimal treatment time and most effective concentration of TGF-β1 for MCs was 24 h and 10 ng/ml respectively [14]. In the experiment, we choose three different concentrations of EP agonists (10, 1, 0.1 μmol/l). And results show that the most effective concentration of the agonists was 1.0 μM. TGF-β1 treatments were generally for 24 h for both RNA analyses and protein analyses.

### RT-PCR for prostaglandin E_2_ receptors

To detect the expression of prostaglandin E_2_ receptors in MCs, we performed RT-PCR. Total RNA was extracted from MCs using the TRI Reagent according to the manufacturer's instructions (Molecular Research Center) and was treated with DNase I to remove contaminating DNA. Two micrograms of RNA was reverse transcribed using Omniscript (Qiagen) in a final volume of 20 ul; this reaction mixture was subsequently divided so that EP_1_, EP_2_, EP_3_ and EP_4_ were amplified from the same reverse transcription mixture using 2 ul in the PCR reaction. Primers for EP_1_, EP_2_, EP_3_ and EP_4_ are described in Table 1. After an initial activation at 94°C for 90 s, PCR was performed for 35 cycles with the following parameters: denaturation at 94°C for 30 s, annealing at 58°C for EP_1_, EP_3_ and EP_4_ and at 66°C for EP_2_ for 1 min, extension at 72°C for 1 min. A final extension was performed at 72°C for 5 min followed by a 4°C hold. Aliquots of the PCR products were then run on a 2% agarose gel in 1× tris-borate EDTA and visualized by ethidium bromide staining and UV illumination.

**Table 1:** Primers used for PCR

	Forward Primer	Reverse Primer
EPI	GCTGCCTATCTTCTCCATGA	TCTGCGCTTGGAGTGATAGA
EP2	ATACTTAGGCCACCGGTCCT	TGAAGCGCATCCTCACAACT
EP3	GCTGCCTATCTTCTCCATGA	GTATACAGGCGAAGCACCAG
EP4	TAGGTCCTGAACATCTGAGGC	CTTGCGCGACTTGCACAATA
COX2	TGAGTACCGCAAACGCTTCT	TCTAGTCTGGAGTGGGAGGC
Mpgesl	GCCTGGTGATGGAGAGCG	AGATGGTGGGCCACCTCC
LN	CTCGTCCCGGTTTCTAAAGG	TTTCCTGCCCTTCTCTCTCA
CTGF	CAAAGCAGCTGCAAATACCA	GGCCAAATGTGTCTTCCAGT
P27	CAGCTTGCCCGAGTTCTACT	TCTGACGAGTCAGGCATTTG
Cyclin Dl	GCGTACCCTGACACCAATCT	CTCTTCGCACTTCTGCTCCT
GAPDH	AGAAGGCTGGGGCTCATTTG	AGGGGCCATCCACAGTCTTC

### Quantitative PCR analysis

Total RNA of MCs was isolated with the RNeasy Plus Mini kit (Qiagen) according to the manufacturer's instructions. Afterward, cDNA was synthesized by iScript reverse transcription (Bio-Rad Laboratories), which was then subjected to SYBR Green-based qRT-PCR using the SYBR Green JumpStart kit (Sigma–Aldrich). PCR reactions were performed and analysed on a Bio-Rad Mini-Opticon detection system, and differences in RNA concentration were controlled by normalizing individual gene signals to those of their corresponding GAPDH reactions. The oligonucleotide primers used are listed in Table 1.

### Western blotting

To examine the expression of cell cycle regulatory molecules we used a Cell Cycle Sampler kit (Cell Signaling Technology) that contains antibodies to all of the molecules (COX-2, mPGES1, P27, cyclin D1, CTGF, LN, P-JNK/p38/ERK and T-JNK/p38/ERK) used in the present study. Proteins were separated by 10% polyacrylamide gel electrophoresis and then transferred on to PVDF membranes (Millipore) at 350 mA for 2 h, which was later soaked for 2 h on a blocking solution (Tris-buffered saline containing 5% nonfat dry milk and 0.01% vol/vol Tween-20). Membranes were incubated overnight at 4°C with rabbit monoclonal primary antibody. After washing with TBS-Tween, membranes were incubated with a horseradish peroxidase-conjugated secondary antibody for 1 h at room temperature. After further washing they were developed using an enhanced chemiluminescent detection method (Amersham Life Science). The membranes were then stripped and re-probed for actin as a loading control.

### Measurement of PGE_2_ in culture medium and intracellular cAMP in MCs

ELISA kits for the detection of cAMP and PGE_2_ were obtained from Sigma and experiments followed the manufacturer's suggested guidelines. Briefly, MCs were cultured in 6-well plates and grown until confluent and subsequently serum deprived for a minimum of 24 h. Cells were then challenged with specified agonist for 30 min prior to the addition of TGF-β1 (10 ng/ml) for 24 h. Culture lysates were then harvested and maintained on ice until the assay was conducted. PGE_2_ and cAMP concentration in the culture medium was measured. Absorbance was measured at 550 nm and data were analysed via interpolation with standards using semilogarithmic paper. A minimum of *n*=3 for each experimental condition was performed.

### Cell cycle analysis

Cells were trypsinized, suspended and washed with PBS. Cell pellets were fixed in 70% ethanol 2 h and then washed with PBS three times. The single-cell suspension was adjusted to 10^7^ cells/ml and stained by propidium iodide staining solution at 4°C for 30 min in the dark. Flow cytometric analysis was performed at a flow rate of 100 events/s using a dual-laser flow cytometer. A total of 10000 events were counted. Cell debris and clumps were excluded from the analysis by gating single cells in the forward and side light scatters. Propidium iodide was excited using the 488-nm UV line of the argon laser. Data were analysed with FCS software (*De Novo* Software).

### Statistical analysis

All experiments were performed in triplicate and the results were expressed as means ± S.E.M. All data were analysed with SPSS 19.0 statistical software using a one-way ANOVA. Two-tailed *P* values of <0.05 were considered statistically significant.

## RESULTS

### Expression of EP receptors in MCs

**Figure 1:**
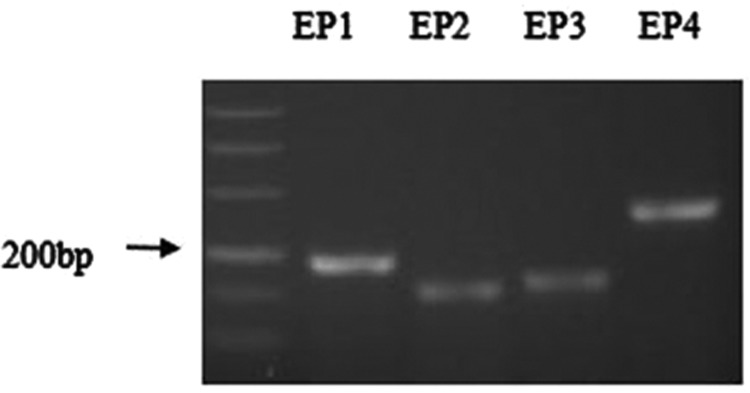
Agarose gel electrophoresis showing RT-PCR products of EP_1_, EP_2_, EP_3_ and EP_4_ receptor expression in cultured mice MCs

The mRNA expression of the four EP receptors in MCs was analysed by RT-PCR. The result (Figure 1) shows all four PGE_2_ receptors (EP receptors) were expressed in mouse MCs.

### Effects of selective EP_1_–EP_4_ receptor agonists on intracellular cAMP levels in mouse MC stimulated with TGF-β1

**Figure 2:**
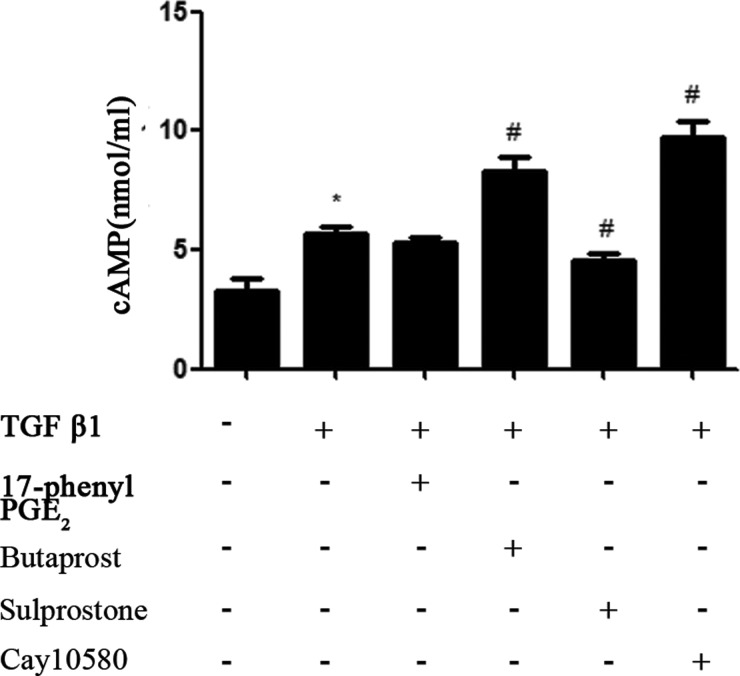
MCs were pre-incubated for 30 min with EP_1_–EP_4_ receptor agonists (1.0 μM) and exposed to TGF-β1 (10 ng/ml) for 10 min Cyclic AMP levels were assessed using cAMP ELISA as described under ‘Experimental Procedures’. Each experimental condition was performed multiple times (*n*=3). Data are expressed as the S.E.M., *P<0.05 compared with Control group, ^#^*P*<0.05 compared with 10 ng/ml TGF-β1 group.

MCs were pre-incubated with EP_1_–EP_4_ receptor agonists for 30 min and exposed to TGF-β1 for 10 min. Then, cAMP production was determined by ELISA. Data demonstrated that TGF-β1 caused a slight increase in cAMP in mouse MCs. EP_1_ agonist treatment showed no significant effects on cAMP level compared with TGF-β1 treated MCs. EP_3_A (1 μM) inhibited TGF-β1-induced cAMP production. In contrast, MCs treated with EP_2_A or EP_4_A increased cAMP levels compared with the control cells (Figure 2). The differences were significant.

### Role of EP receptors in the regulation of TGF-β1-induced expression of COX-2 and mPGES1 and secretion of PGE2 in mouse MC

**Figure 3:**
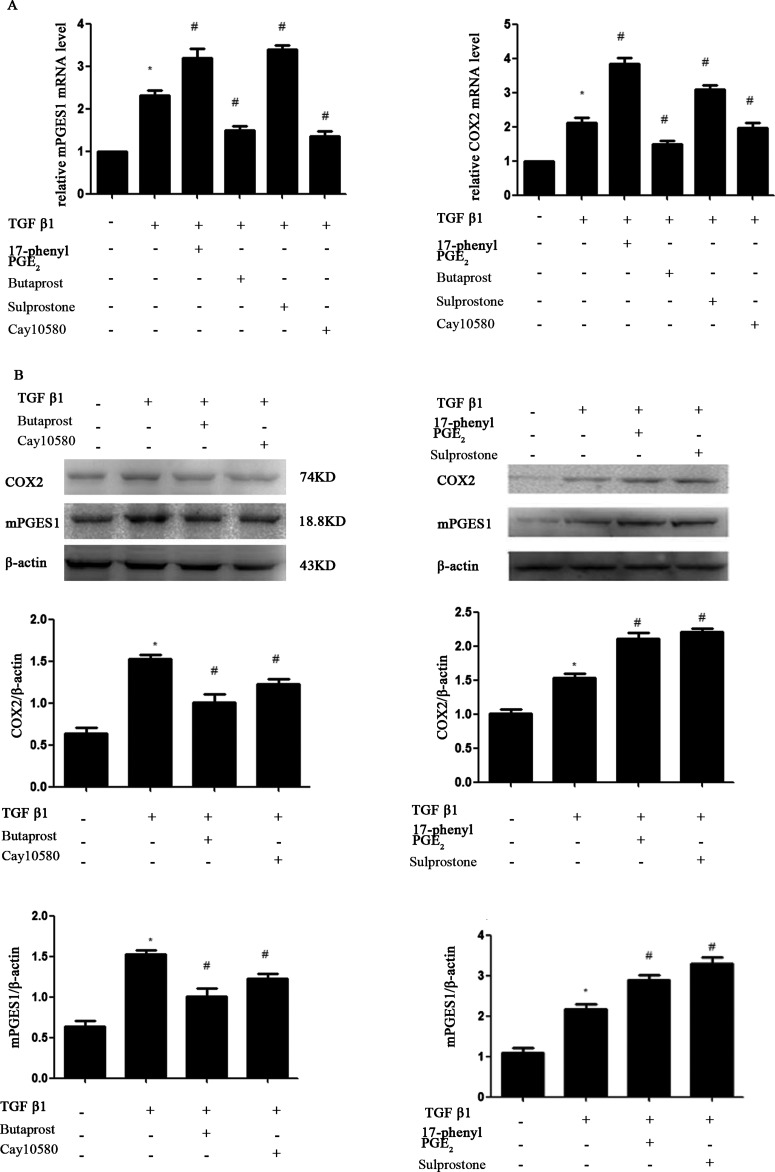
MCs cultures were pretreated for 30 min with EP_1_–EP_4_ receptor agonists (1.0 μM), respectively, prior to the addition of 10 ng/ml TGFβ1 Cultures were incubated for an additional 24 h prior to harvest of total protein and mRNA for qPCR and Western blot. Western blot and RT-PCR analysis were used to determine the expressions of COX-2, mPGES1. (**A**) RT-PCR; (**B**) Western blot. Each experimental condition was performed multiple times (*n*=3). Data are expressed as the S.E.M., *P<0.05 compared with Control group, ^#^*P*<0.05 compared with 10 ng/ml TGF-β1 group.

To determine the effect of EP receptors on the prostanoid synthetic pathway, we used real-time quantitative RT-PCR and western blotting to compare mRNA and protein abundance of COX-2 and mPGES1 in each group. MCs were pre-incubated with EP_1-4_ receptor agonists for 30 min and exposed to TGF-β1 for 24 h. As shown in Figure 3, TGF-β1 induced the expression of COX-2 and mPGES1 mRNA as well as protein. Compared with TGF-β1 treated MCs, the expression of COX-2 and mPGES1 in MCs treated with EP_2_A or EP_4_A was significantly suppressed. In contrast, there was a significant elevation in COX-2 and mPGES1 expression in MCs treated with EP1 or EP_3_ receptor agonists.

**Figure 4:**
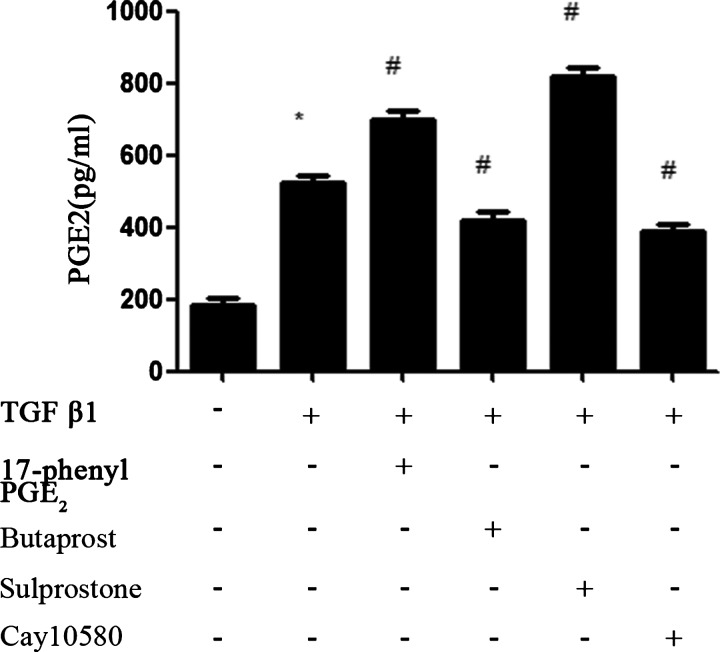
Production of PGE_2_ in culture supernatants was determined by ELISA MCs cultures were pretreated for 30 min with EP_1_–EP_4_ receptor agonists (1.0 μM), respectively, prior to the addition of 10 ng/ml TGFβ1 for an additional 24 h. Each experimental condition was performed multiple times (*n*=3). Data are expressed as the S.E.M., *P<0.05 compared with Control group, ^#^*P*<0.05 compared with 10 ng/ml TGF-β1 group.

We next assessed whether the change in COX-2 and mPGES1 expression has a functional consequence such as an adjustment in prostanoids production. We then examined the production of PGE_2_ by ELISA in culture supernatants. Our results suggested that PGE_2_ production was consistent with the increase in COX-2 and mPGES1 expression (Figure 4).

### Effects of selective EP_1_–EP_4_ receptor agonists on TGF-β1-induced expression of mRNA and protein of LN and CTGF

**Figure 5:**
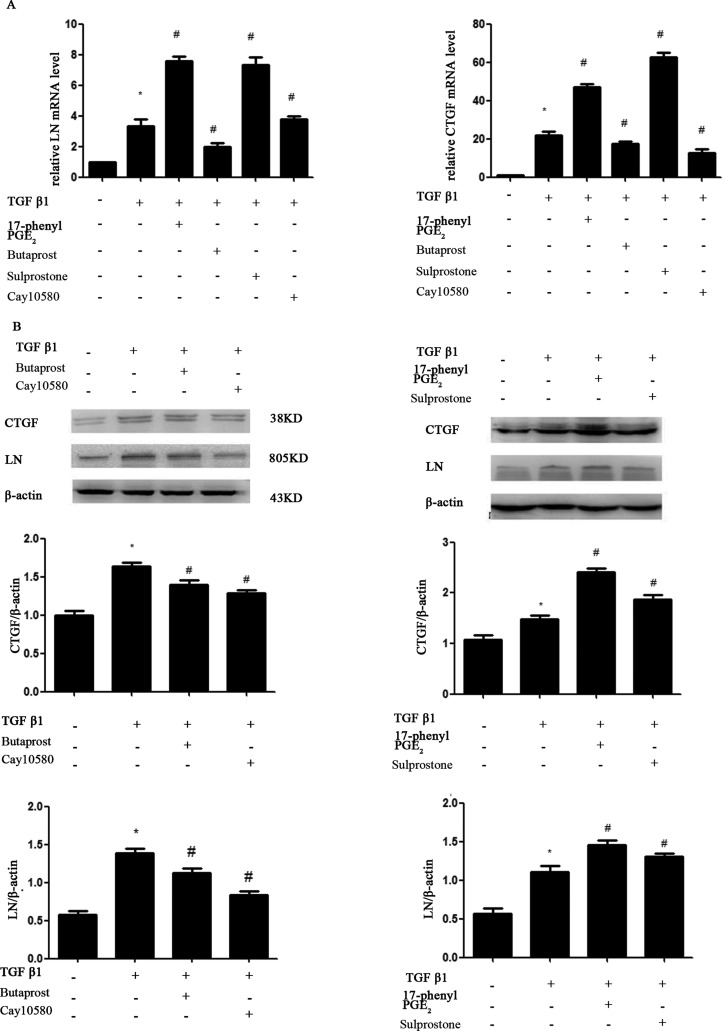
MCs cultures were pretreated for 30 min with EP_1_–EP_4_ receptor agonists (1.0 μM), respectively, prior to the addition of 10 ng/ml TGF-β1 for an additional 24 h Total protein and mRNA were harvested for RT-PCR and Western blot. Western blot and RT-PCR analysis were used to determine the expressions of LN and CTGF. (**A**) RT-PCR; (**B**) Western blot. Each experimental condition was performed multiple times (*n*=3). Data are expressed as the S.E.M., *P<0.05 compared with Control group, ^#^*P*<0.05 compared with 10 ng/ml TGF-β1 group.

Published reports have shown that TGF-β1 also plays a major role in glomerular ECM accumulation in several glomerular diseases [15]. Because PGE_2_ levels are increased in MCs, we suspected that accumulation of ECM induced by TGF-β1 may be due to the increased PGE_2_ levels. We therefore investigated the role of EP receptors in the regulation of accumulation of ECM. MCs were pre-incubated with EP_1_–EP_4_ receptor agonists for 30 min and exposed to TGF-β1 for 24 h. As shown in Figure 5, TGF-β1-induced expression of LN and CTGF was inhibited by EP_2_A and EP_4_A, but enhanced by EP_1_ or EP_3_ receptor agonists in MCs.

### Effect of four selective EP_1_–EP_4_ receptor agonists on MC cycle and TGF-β1-induced expression of p27kip1 and cyclin D1

**Figure 6:**
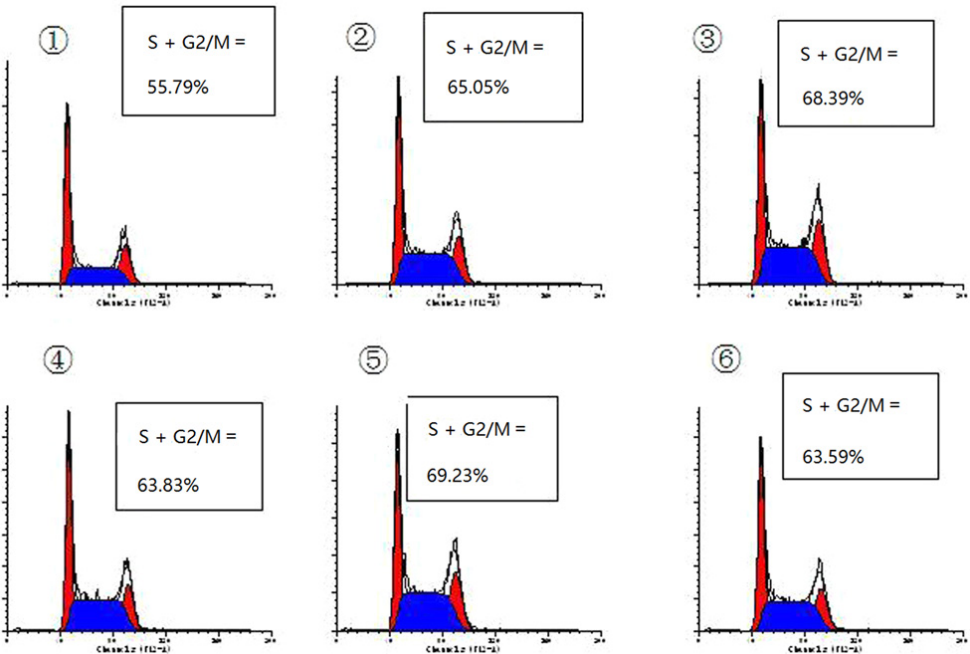
Cell cycle analysis of MCs assessed by fluorescence-activated cell-sorting analysis After pre-incubation in DMEM containing 0.5% FCS for 24 h, growth-arrested cells were pre-treated with or without EP_1_–EP_4_ receptor agonists (1.0 μM) and the co-incubation was continued for 24 h after the addition of TGF-β1 (10 ng/ml) in DMEM containing 0.5% FCS. Cell cycle analysis assessed by the incorporation of propidium iodide into DNA by fluorescence-activated cell-sorting analysis using a dual laser flow cytometer. ① Cells were treated with 0.5% FCS alone. ② Cells were treated with TGF-β1 alone for 24 h. ③ Cells were treated with TGF-β1 (10 ng/ml) for 24 h with pre-incubation of 17-phenyl PGE_2_(1.0 μM) for 30 min. ④ With pre-incubation of butaprost(1.0 μM) for 30 min. ⑤ With pre-incubation of sulprostone (1.0 μM) for 30 min. ⑥ With pre-incubation of cay10580 (1.0 μM) for 30 min. Data are S.E.M. of three independent experiments.

**Figure 7:**
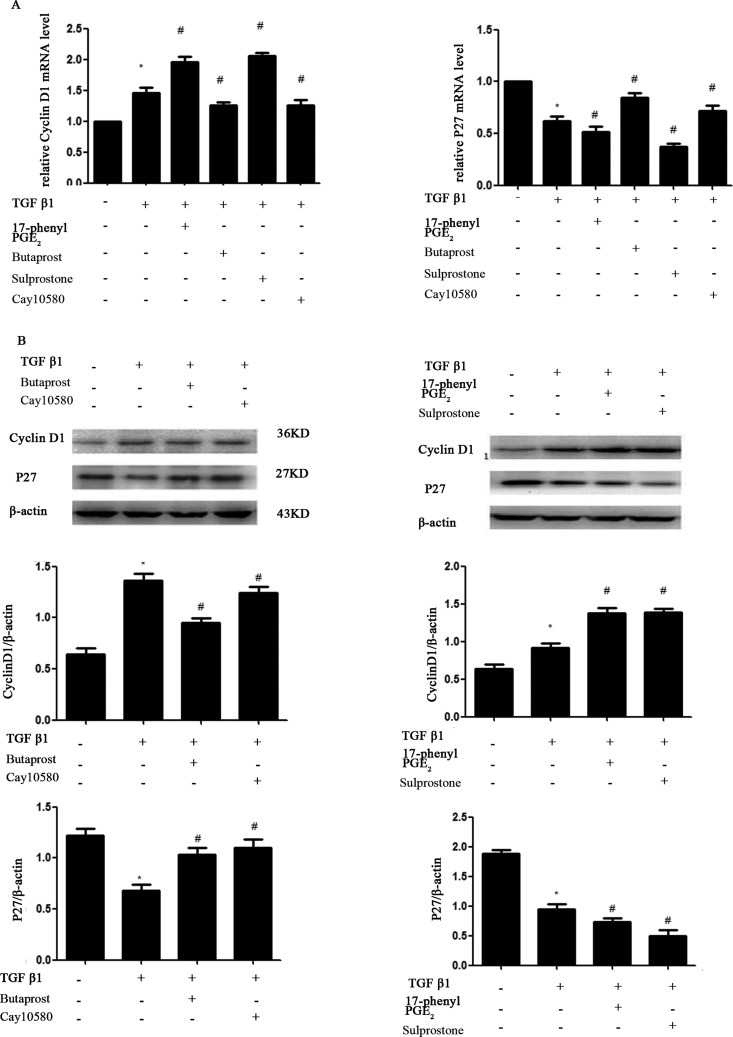
Western blot and RT-PCR analysis of p27kip1 and cyclin D1 Cultured and serum-deprived cells were treated with TGF-β1 (10 ng/ml) in DMEM containing 0.5% FCS for 24 h and pre-treatment of EP_1_–EP_4_ receptor agonists (1.0 μM) for 30 min. The expressions of p27kip1 and cyclin D1 were detected with Western blot and RT-PCR. Actin served as a loading control. (**A**) RT-PCR; (**B**) Western blot. Each experimental condition was performed multiple times (*n*=3). Data are expressed as the S.E.M., *P<0.05 compared with Control group, ^#^*P*<0.05 compared with 10 ng/ml TGF-β1 group.

Figure 6 shows the effects of TGF-β1 and four EP agonists on the cell cycle phase distribution of MCs. The results showed that treatment with TGF-β1 (10 ng/ml) for 24 h resulted in cell accumulation in S+G2/M phase and reduction in G0/G1 phase, compared with the control cells. Pre-treatment with EP_2_A or EP_4_A (1.0 μM), most cells were arrested in G0/G1 phase, whereas pre-treatment with EP_1_A or EP_3_A (1.0 μM) were manifesting just the opposite way. The results of western blotting showed that TGF-β1 decreased the expression of p27kip1 (*P*<0.05). The p27kip1 protein level increased in MCs after EP_2_A or EP_4_A treatment, whereas the protein level decreased in EP_1_A or EP_3_A treated MCs (*P*<0.05). And the results of western blotting showed that TGF-β1 increased the expression of cyclin D1 (*P*<0.05). The expression levels of cyclin D1 were down-regulated in MCs treated with EP_2_A or EP_4_A, whereas the expression of cyclin D1 was up-regulated in MCs treated with EP1A or EP_3_A (*P*<0.05) (Figure 7).

### Multiple MAPK routes participate in PGE_2_-induced proliferation and extracellular matrix synthesis in mouse MC stimulated with TGF-β1

**Figure 8:**
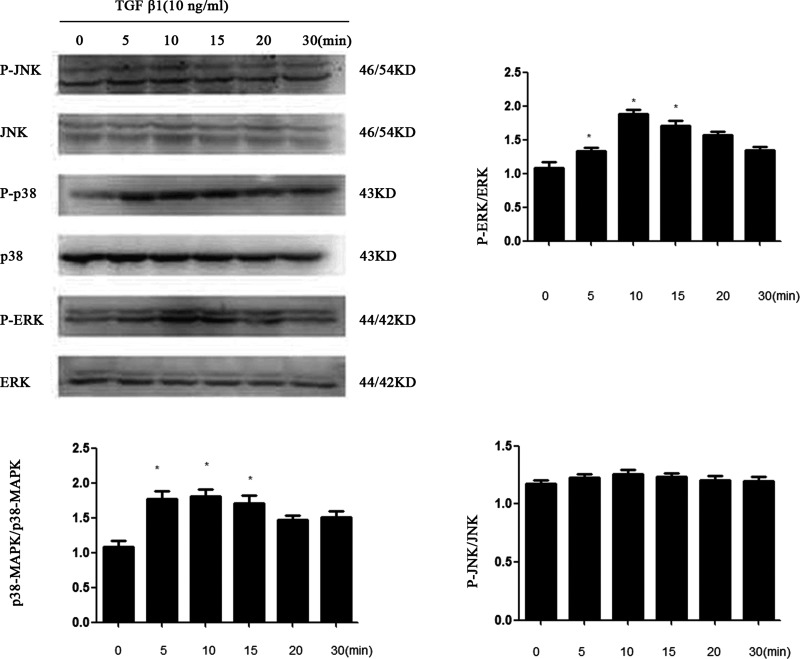
MCs were serum-starved for 24 h and then stimulated with TGF-β1 (10 ng/ml) for the indicated time periods Total protein extracts were analysed by Western blotting using specific antibodies for total and phosphorylated ERK1/2, p38 MAPK, JNK (*P<0.05 compared with 0 h). P-JNK/p38/ERK: phosphorylated JNK, phosphorylated p38, phosphorylated ERK. T-JNK/p38/ERK: Total JNK, Total p38, Total ERK.

**Figure 9:**
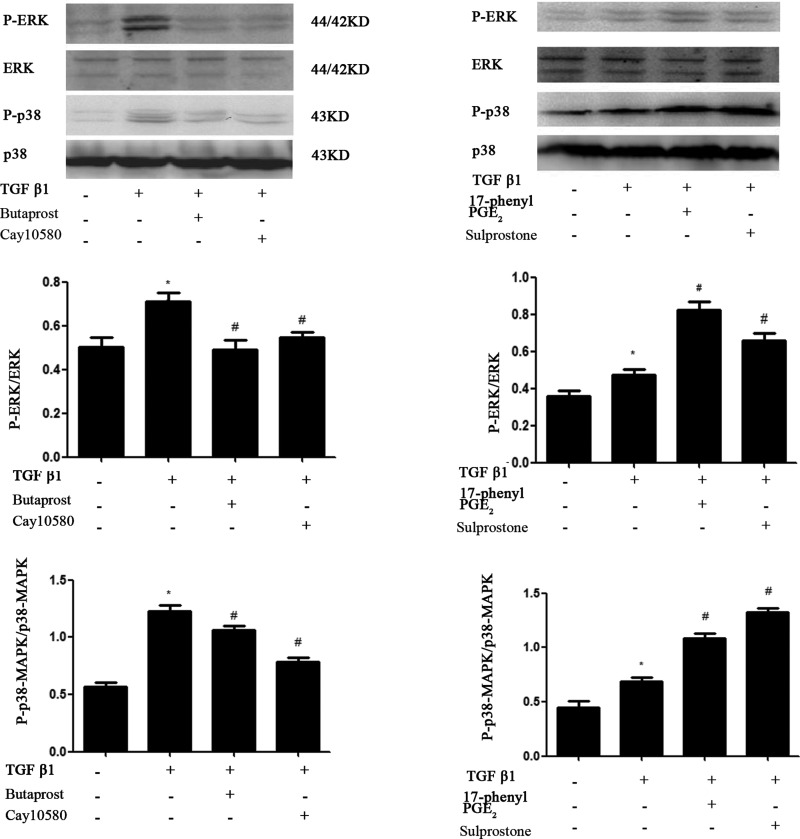
MCs were serum-starved for 24 h and pretreated with 17-phenyl trinor PGE_2_ (1.0 μM), butaprost (1.0 μM), sulprostone (1.0 μM) and cay10580 (1.0 μM) for 30 min before stimulation with TGF-β1 (10 ng/ml) for 10 min Total protein extracts were analysed by Western blotting using specific antibodies for total and phosphorylated ERK1/2, p38 MAPK, phosphorylated ERK1/2, p38 MAPK protein expression levels were analysed by Western blotting. Data are expressed as the S.E.M. (*P<0.05 compared with Control group, #*P*<0.05 compared with 10 ng/ml TGF-β1 group).

Western blot analysis demonstrated that TGF-β1 significantly increased the level of the phosphorylated forms of ERK1/2 and p38 but not JNK1/2 in MCs and reached a maximum after 10 min of treatment of TGF-β1 and then decreased gradually. Total ERK1/2 remained constant throughout the duration of the experiment (Figure 8). To determine which receptor is involved in TGF-β1-induced ERK1/2 and p38 phosphorylation in MCs, cells were pre-treated with the EP_1_–EP_4_ receptor agonists and then treated with TGF-β1 for 10 min. EP_2_ and EP_4_ agonist considerably attenuated TGF-β1-induced ERK1/2, p38 phosphorylation, whereas EP_1_ and EP_3_ agonists increased their phosphorylation (Figure 9).

## DISCUSSION

PGE_2_, a major PG in the kidney, plays an important role in renal physiology, including the regulation of vascular smooth muscle tonus, glomerular filtration, renin release and tubular salt and water transport [4]. The large variety of biological functions governed by prostaglandin E2 is mediated by signalling through four distinct E-type prostanoid receptors [16]. Although the regulation mechanism of PGE_2_ on salt and water metabolism under physiological conditions are understood, our understanding of how PGE_2_ influence Na or sodium homoeostasis and blood pressure regulation under pathological conditions is in its infancy [17]. Makino et al. [18] has shown that an EP_1_ antagonist, and a nonselective PG synthase inhibitor, markedly attenuate mesangial expansion, inhibits glomerular hypertrophy and proteinuria of diabetic nephropathy in rats. Black et al. [19] found that stimulation of the EP_3_ receptor with sulprostone resulted in a robust increase in TGF-β1-stimulated CTGF levels in human renal MCs. We speculate that the COX-2–PGE_2_–EP system might play an important role in the renal fibrosis process.

The cDNA for these receptors have been cloned, their signal transduction mechanisms determined, and their intrarenal distribution mapped. Four distinct EP receptors are highly expressed in specific regions of the kidney [4]. In the present paper, EP_1_, EP_2_, EP_3_ and EP_4_ receptors are expressed in the mouse MCs by RT-PCR, although the expression of EP_4_ and EP_2_ are relatively lower than EP_3_ and EP_1_.

TGF-β is a cytokine known to participate in several processes related to the development of CKD [20,21]. TGF-β is known to induce the expression of COX-2, mPGES1 and PGE_2_ in MCs [14]. The COX-2 inhibitor etodolac significantly reduces TGF-β, resulting in decreased tubular damage and interstitial fibrosis [22]. What are the four EP receptors' role in the process of renal tubular damage and interstitial fibrosis? We designed studies to determine whether TGF-β1-induced expression of COX-2, mPGES1 and PGE_2_ in MCs is affected by four EP receptor agonists. We demonstrate that 17-phenyl PGE_2_(EP_1_A)and sulprostone(EP_3_A) increased the expression of COX-2, mPGES1 and PGE_2_ whereas butaprost(EP_2_A)and cay10580 (EP_4_A) decreased their expression. LNs are major proteins in the basal lamina. They are an important and biologically active part of the basal lamina, influencing cell differentiation and migration. CTGF is an established effector of TGF-β1 driven responses in diabetic nephropathy. We next obtained that TGF-β1 stimulated LN and CTGF expression in MCs. Activation of EP_1_ and EP_3_ by 17-phenyl PGE_2_ and Sulprostone notably increased the TGF-β1-induced LN and CTGF protein expression in MCs, whereas EP_2_A and EP_4_A inhibited their expression. We demonstrate that EP_1_A and EP_3_A up-regulate LN and CTGF expression in MCs through the activation of COX-2-PGE_2_, which lead to MCs damage. EP_2_ and EP_4_ agonists elicit opposite responses. All these data suggest that PGE_2_ via EP_2_ and EP_4_ may slow the progression of renal fibrosis whereas EP_1_ and EP_3_ may promote the process of renal damage.

Among many factors that regulate matrix deposition in different ways, TGF-β1 is the most potent inducer that is capable of initiating and completing the entire matrix deposition course. However, TGF-β1 may also have other important functions in the glomerulus, including the regulation of MCs proliferation [23]. Cell cycle progression is driven by the coordinated regulation of the activities of cyclin, cyclin-dependent kinases (Cdks) and Cdk-inhibitor (CKI) [24]. CKIs are regulated by intracellular signalling pathways mediated by extracellular growth factors [25]. p27Kip1 blocks the catalytic activity of cyclin/Cdk complexes, inducing cell cycle arrest in mid-G1 phase [26]. Cyclin D1 involved in the regulation of cell cycle from G1 to S phase. In our study, TGF-β1 increased the proportion of MCs arrested in G2/S phase and the proliferation of cyclin D1, whereas down-regulated the expression of P27 protein and mRNA. EP_1_A and EP_3_A promoted TGF-β1-induced MCs proliferation, whereas EP_2_A and EP_4_A alleviated such effects of TGF-β1 on MCs. Thus, the above results suggest that increased production of PGE_2_ induced activation of EP_1_ and EP_3_, which then led to TGF-β1-induced changes of cell proliferation and cell cycle. Whereas EP_2_ and EP_4_ are important in preserving the cell proliferation.

However, what are the molecular mechanisms underlying the effects of EP_1_–EP_4_ on TGF-β1-induced MCs damage? MAPK families play an important role in complex cellular programs like proliferation, differentiation, development, transformation, and apoptosis. At least three MAPK families have been characterized: extracellular signal-regulated kinase (ERK), Jun kinase (JNK/SAPK) and p38 MAPK [27–29]. Specific activation of MAPK pathways may mediate TGF-β1-induced cell or tissue effects. Therefore, our study mainly focused on the interaction between cAMP and MAPK signalling pathways. We found that ERK and p38 were activated obviously after stimulation of MCs with TGF-β1 for 10 min whereas this treatment showed no significant effects on JNK levels. EP_1_ agonist treatment showed no significant effects on cAMP levels, but the expression of p-p38 and p-ERK was increased. Furthermore, cAMP production in response to TGF-β1 was markedly reduced in MCs treated with EP_3_ agonist whereas ERK and p38 phosphorylation was increased. The level of TGF-β1-dependent cAMP production was increased with EP_2_ or EP_4_ agonist treatment whereas ERK and p38 phosphorylation was decreased. Taken together, these results demonstrate that EP_1_ agonist can activate p38MAPK and ERK phosphorylation by regulating gated calcium channel through G protein. EP_3_ agonist blocked cAMP production, at least in part, by inhibiting adenylate cyclase activity through inhibitory G protein receptor and resulted in activation of p38MAPK and ERK phosphorylation. Such reactions are critical mediators of renal injury by promoting MCs proliferation and ECM fibrosis. By contrast, EP_2_ and EP_4_ agonists showed the opposite effects on TGF-β1-induced MCs injury. It is indicating that inhibition of p38MAPK and ERK phosphorylation is dependent on cAMP production [30–32].

Although 17-phenyl PGE_2_ and sulprostone can activate EP_1_ and EP_3_ receptors simultaneously, their G protein-coupled receptors and specific downstream effectors are different. But both of EP_1_ and EP_3_ can activate the MAPKS signalling pathway and induce p38MAPK and ERK phosphorylation. Our results provide evidence that EP_1_ and EP_3_ receptors may play an important role in TGF-β1-induced MCs injury. Furthermore, we showed that when MCs were treated with butaprost or cay10580, the levels of cell fibrosis markers were significantly decreased, suggesting that EP_2_ and EP_4_ agonists can prevent the development of TGF-β1-induced MCs injury, these data were in agreement with those of Zahner et al. [33,34]. However, Mohamed et al. [35] found that EP_4_ agonist through IL-6 induced glomerulosclerosis and interstitial fibrosis of kidneys in an animal model of type 1 diabetes. Whether the contradiction between the two results was ultimately due to different agonist concentration or differences in actual action time of EP_4_ or tissue difference or any other reasons should be further studied. Furthermore, it may be due to the dual role of PGE_2_.

In conclusion, our results demonstrate that in cultures of MCs, TGF-β1 induces cell proliferation and the production of ECM, increase the expression of COX-2, mPGES1 and PGE_2_. EP_1_ and EP_3_ agonists both aggravated TGF-β1-mediated renal injury by promoting the activation of p38MAPK and ERK phosphorylation. In contrast, EP_2_ and EP_4_ agonists inhibited TGF-β1-induced cell proliferation by inhibiting downstream targets of kinase cascade, such as cell cyclin and CTGF, through inhibition of MAPKs signalling pathway. These results may open up specific strategies for the treatment of renal glomerular inflammatory diseases that specifically target the pathways involved in EP receptor activation.

## Abbreviations

Cdk: cyclin-dependent kinase
CKD: chronic kidney diseases
CKI: CDK-inhibitor
COX-2: cyclooxygenase-2
CTGF: connective tissue growth factor
ECM: extracellular matrix
ERK1/2: extracellular-signal-regulated kinase 1/2
FCS: foetal calf serum
LN: laminin
MC: mesangial cell
mPGES1: membrane-bound PGE synthase 1
PI: phosphatidylinositol
